# Measles Still Has a Devastating Impact in Unvaccinated Populations

**DOI:** 10.1371/journal.pmed.0040024

**Published:** 2007-01-02

**Authors:** William J Moss

## Abstract

Although global mortality from measles is falling, a study in*PLoS Medicine* found that children in Nigeria, Niger, and Chad face unacceptably high mortality from measles, a largely preventable disease.

Measles has caused millions of deaths since its emergence thousands of years ago, probably as a zoonosis [1]. Deaths from measles are due largely to an increased susceptibility to secondary bacterial and viral infections. This period of increased susceptibility lasts for several weeks to months after the onset of rash [2] and is attributed to a prolonged state of immune suppression. Most deaths associated with measles are due to pneumonia [3]. Although the global mortality from measles is falling [4], a new study in *PLoS Medicine* found that children in Nigeria, Niger, and Chad still recently faced unacceptably high mortality from measles [5], a largely preventable disease.

## Measles Case Fatality Ratios

Measles case fatality ratios vary depending upon the average age of infection, the nutritional status of the population, measles vaccine coverage, and access to health care. In developed countries, less than one in 1,000 children with measles die. But in endemic areas in sub-Saharan Africa, the measles case fatality ratio often ranges from 5%–10%.

Measles is a major cause of child death in refugee camps and in internally displaced populations, and case fatality ratios in children in complex emergencies have been as high as 20%–30%. For example, during a famine in Ethiopia, measles alone or in combination with wasting accounted for 22% of 159 deaths among children younger than five years of age, and 17% of 72 deaths among children aged five to 14 years [6].

The risk of death following measles is highest at extremes of age and in those with underlying malnutrition and vitamin A deficiency. Exposure to an index case within the household may result in more severe disease, perhaps because of transmission of a larger inoculum of virus [7].

Interestingly, data suggest that measles mortality may be higher in girls. Among persons of different ages and across different regions (primarily in the Americas and Europe), measles mortality in girls was estimated to be 5% higher than in boys [8].

Supporting the hypothesis of biological differences in the response to measles virus between boys and girls was the observation that girls were more likely than boys to have delayed mortality following receipt of high-titer measles vaccine [9].

## Measles Vaccination

Vaccinated children, should they develop disease after exposure, have less severe disease and significantly lower mortality rates. Vaccination programs, by increasing the average age of infection, shift the burden of disease out of the age group with the highest case fatality (infancy), further reducing measles mortality.

Several attenuated measles vaccines are available worldwide, either as single-virus vaccines or in combination with other vaccine viruses (commonly rubella and mumps). The proportion of children who develop protective antibody levels following measles vaccination depends on the presence of inhibitory maternal antibodies and the immunological maturity of the vaccine recipient, as well as the dose and strain of vaccine virus. About 85% of children develop protective antibody levels when the measles vaccine is administered at nine months of age, and 90%–95% have a protective antibody response after vaccination at 12 months of age [10]. Most children who do not respond to a first dose of measles vaccine will respond to a second dose at an older age. The duration of protective antibody levels following measles vaccination is more variable and shorter than that acquired through infection with wild-type measles virus, with an estimated 5% of children losing protective antibody levels 10 to 15 years after vaccination [11].

## The Global Decline in Measles Mortality

Prior to the development and widespread use of measles vaccines, measles was estimated to result in 5 to 8 million deaths annually. The decline in mortality from measles in developed countries was associated with economic development, improved nutritional status and supportive care, and antibiotic therapy for secondary bacterial pneumonia, as well as the widespread use of measles vaccine.

In 2003, the World Health Assembly endorsed a resolution urging member countries to reduce the number of deaths attributed to measles by 50% by the end of 2005 compared with 1999 estimates, a target that is likely to have been met. Overall global measles mortality in 2004 was estimated to be 454,000 deaths (uncertainty bounds 329,000 and 596,000 deaths), a 48% decrease from 1999 [4]. Following this success, the global measles control goal now is to reduce the annual number of measles deaths by 90% by 2010 compared to the estimated number in 2000.

Remarkable progress in reducing measles incidence and mortality has been made in parts of sub-Saharan Africa as a consequence of increasing measles vaccine coverage, provision of a second opportunity for measles vaccination through supplementary immunization activities, improved case management, and enhanced surveillance with laboratory confirmation of measles cases [12]. This accelerated measles control strategy began in 2001 with the support of the Global Alliance for Vaccines and Immunization (http://www.gavialliance.org) and the Measles Initiative (http://www.measlesinitiative.org).

## Recent Outbreaks in Three African Countries: A New Study

How successful has this accelerated measles control strategy been? One measure of success is the recent demonstration of measles outbreaks in countries where these strategies had *not* been implemented.

The new study in *PLoS Medicine*, by Rebecca Grais and colleagues, provides estimates of measles attack rates and age-specific case fatality ratios during large measles outbreaks in Niger, Chad, and Nigeria during 2004–2005 [5]. The authors conducted retrospective household surveys in one neighborhood of each affected area at a time when the measles outbreak was subsiding, encompassing a total population surveyed of over 64,000 individuals. Routine measles vaccine coverage rates were low, supplementary immunization activities had not yet been conducted, access to health care was poor, and responses to the outbreaks were delayed, an epidemiological situation conducive to the rapid spread of measles virus with high rates of morbidity and mortality.

Almost 3,200 cases of measles were identified in the study areas with age-specific attack rates of 17% to 24% in children less than five years of age. About two-thirds of persons with measles developed respiratory complications and diarrhea. The case fatality ratio ranged from 2.8% to 7% and was highest in children less than five years of age. Most deaths occurred in unvaccinated children and at home. In rural Nigeria the risk of death was higher in girls than boys.

Although subject to potential recall and misclassification biases, the estimates of morbidity and mortality during these large outbreaks of measles in Niger, Chad, and Nigeria are consistent with prior estimates obtained in similar epidemiological settings. The population surveyed was large and the non-participation rate was small.

## Implications of the Study

At a time of accelerated measles control in sub-Saharan Africa, these estimates serve as a reminder of the devastating impact measles can have in unvaccinated populations. During the outbreak, half of all deaths in children less than five years of age were attributed to measles. Prompt recognition and response to measles outbreaks, in addition to appropriate case management, is critical to reducing measles morbidity and mortality and preventing further transmission of measles virus.

These outbreaks also should serve as a reminder to communities in resource-rich countries of the importance of measles vaccination and the potential for imported measles cases to spark outbreaks in unvaccinated communities. Following the reported outbreaks in Niger, Chad, and Nigeria, accelerated measles control strategies were implemented in the affected areas, including mass measles vaccination campaigns. However, only sustained efforts to maintain high coverage rates of the routine first dose of measles vaccine, coupled with periodic opportunities for a second dose, will achieve the level of population immunity required to avert the unacceptably high morbidity and mortality rates that result from measles epidemics in susceptible populations.

## 

**Figure:**
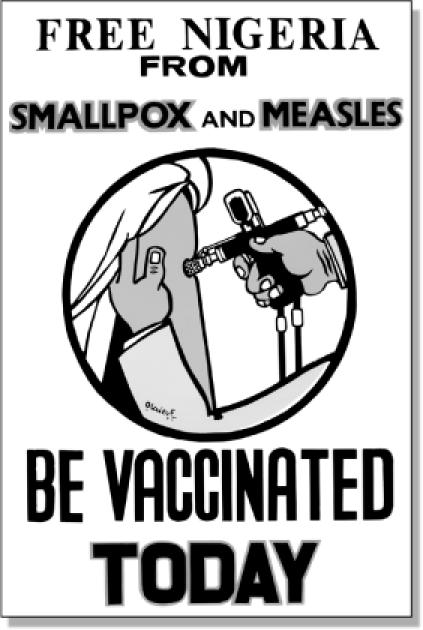
A pre-1979 poster from Nigeria promoting vaccination for smallpox and measles (image: CDC/Stafford Smith).
